# An Ensemble Multilabel Classification for Disease Risk Prediction

**DOI:** 10.1155/2017/8051673

**Published:** 2017-06-15

**Authors:** Runzhi Li, Wei Liu, Yusong Lin, Hongling Zhao, Chaoyang Zhang

**Affiliations:** ^1^Cooperative Innovation Center of Internet Healthcare, Zhengzhou University, Zhengzhou, China; ^2^School of Computing, University of Southern Mississippi, Hattiesburg, MS, USA

## Abstract

It is important to identify and prevent disease risk as early as possible through regular physical examinations. We formulate the disease risk prediction into a multilabel classification problem. A novel Ensemble Label Power-set Pruned datasets Joint Decomposition (ELPPJD) method is proposed in this work. First, we transform the multilabel classification into a multiclass classification. Then, we propose the pruned datasets and joint decomposition methods to deal with the imbalance learning problem. Two strategies size balanced (SB) and label similarity (LS) are designed to decompose the training dataset. In the experiments, the dataset is from the real physical examination records. We contrast the performance of the ELPPJD method with two different decomposition strategies. Moreover, the comparison between ELPPJD and the classic multilabel classification methods RAkEL and HOMER is carried out. The experimental results show that the ELPPJD method with label similarity strategy has outstanding performance.

## 1. Introduction

To identify and prevent chronic diseases as early as possible is significant. Machine learning can discover hidden knowledge from a huge amount of disease-related data. It is feasible to construct models for prediction of disease risk based on big physical examinations data. There are two classic types of classifiers for the supervised learning: single-label classification and multilabel classification, which are based on the number of class labels of each record. In single-label classification, where classes are mutually exclusive by definition, each instance is associated with one class label. However, multilabel classification means one instance corresponds to more class labels. In medical diagnosis, a symptom may belong to multiple disease types. How to simultaneously predict the risk of several normal chronic diseases remains a challenging problem.

In this work, we focus on the multilabel disease risk prediction and high accuracy on the prediction results based on physical examinations. The proposed method is called ELPPJD (Ensemble Label Power-set Pruned datasets Joint Decomposition), which transforms the multilabel classification into multiclass classification by an improved label power-set method. The pruned technique is used to balance the size of multilabel combination labels. We present a novel partition strategy to decompose the training dataset based on label similarity among labels. A large number of experiments are conducted to compare with other methods. Empirical evidences consisting of accuracy, precision, recall, and F measure indicate that proposed ELPPJD has better performance than others for disease risk prediction based on physical examinations.

The traditional healthcare industry is undergoing a major paradigm shift due to the rapid advances and developments in mobile and other wireless technologies, which brings big benefits to the health information management and prevention of human disease. Based on this work, we can provide individual health risk self-examination by developing intelligent mobile applications. Once individual physical examination outcomes are uploaded, multiple disease risks could be predicted based on these intelligent mobile applications.

## 2. Related Works

There is plenty of literature that analyzes or predicts the risk of one single disease at a time. For example, Yeh et al. [1], Shivakumar and Alby [2], and Neuvirth et al. [3] focused on diabetes analysis. The models were built for predicting the cerebrovascular disease [1, 4]. These predictions of single diseases are formulated into the binary classification problems. However, multiple-related diseases may appear simultaneously, where binary classification cannot deal with it effectively. In this work, we focus on formulating multilabel classification to resolve the multidisease risk prediction based on physical examination records.

Matthew et al. [5] described firstly the multilabel classification and defined the multilabel classification tasks in which some instances belong to multiple classes. Tsoumakas et al. [6] summarized two multilabel classification methods consisting of algorithm adaptation and problem transformation. The algorithm adaptation methods mean that they can be adapted, extended, and customized to solve the multilabel learning tasks based on the basic machine learning algorithms. ADABOOST.MH and ADABOOST.MR [7] are two extensions of ADABOOST based on boosting. ML-KNN is another algorithm adaptation method, which based on k-Nearest Neighbors (kNN) [8]. ML-C4.5 algorithm also is an algorithm-adapted method based on C4.5 algorithm by modifying the formula to calculate entropy. The problem transformation methods belong to another type of multilabel classification. They transform the multilabel learning problem into a single-label classification problem. Here are three types of classic problem transformation methods: binary relevance (BR), label power-set (LP), and pair-wise. Binary relevance uses the one-against-all idea to convert the multilabel problem into |*L*| binary classification problems, where |*L*| denotes the size of label set [9]. However, BR suffers from the label independence assumption, and it fails to utilize any relationships between labels. Label power-set methods [9] transform a multilabel problem into a single-label problem by transforming each instance's label set *S_i_* to an atomic label *l*_*i*_′. For example, the multilabel set a,c,d would become a single label acd. LP overcomes the label independence assumption problem, but would suffer the worst-case time complexity when the size of single label-set is large. Pair-wise methods [10] use round robin classification with binary classifiers to solve the multilabel problem. It constructs Q.(Q-1)/2 classifiers to cover all pairs of labels, which adopt the majority voting algorithm to combine all the classifiers. There are some improved methods proposed in some literatures. Read et al. [11] presented a Pruned Sets method (PS), which focuses on core relationships within multilabel sets through pruning labels that occur less than a predefined number of times. This method would reduce the complexity to deal with a large number of infrequent sets. Random k-label sets (RAkEL) is an ensemble method, which constructs each base classifier by considering a small random subset of labels and learning a single-label classifier for the prediction of each element in the power-set of this subset [12]. HOMER [13] is another extended label power-set method, which constructs a hierarchy tree of label sets. Every node would produce a classifier in the hierarchy tree. In RAkEL and HOMER methods, multiple classifiers are constructed. Classifier chains ensemble method is used for classification and prediction, where the final prediction is obtained by summing the predictions by label and applying threshold for selecting the relevant labels [14]. Madjarov et al. [15] presented an extensive experimental comparison, which use 12 multilabel learning methods on 16 evaluation measures over 11 benchmark datasets.

In summary, different multilabel learning methods have merits and demerits, which depend on specific applications. It still remains a challenging problem to accurately and efficiently predict the health or disease risks based on the medical records. This work is an extension of work originally presented in conference 2016 BIBM [16]. A multilabel classification method MLP-TJC was proposed. However, it gives poor prediction accuracy for the infrequent multilabel classes and also lacks comprehensive evaluations. In this work, we improve the previous multilabel method and develop a novel ensemble method ELPPJD. In the experiments, we expand the numbers of single labels and combination labels. We also compare the ELPPJD with other outstanding multilabel classification methods.

## 3. Problem Statement

An example of multilabel physical examination records is shown in Table 1. The disease risk prediction is formulated into a multilabel classification problem. Given a set of *m* medical records *D* = {*r*_1_,…, *r*_*m*_}, with *r_i_*, *i* = 1,…, *m*, and a set of *n* disease labels *L* = {*l*_1_,…, *l*_*n*_}, with *l_j_, j* = 1,…, *n*, denoting one type of disease, each record in *D* is associated with one or more disease labels in *L*. The problem of multilabel disease classification can be represented by a tuple of (*r_i_*, *S_i_*), where *S_i_* is the class label for record *r_i_. S_i_* is a subset of *L*, which denotes *S*_*i*_⊆*L*. Our objective is to construct a classification model to predict the disease label *S*_*i*_′ for every new physical record *r*_*i*_′.

## 4. An Ensemble Multilabel Classification Method

We propose a novel ensemble multilabel classification method ELPPJD (Ensemble Label Power-set Pruned datasets Joint Decomposition) for the disease risk prediction based on the physical examination records. It transforms a multilabel classification problem into a multiclass classification problem. The idea comes from the classic label power-set (LP) method, which overcomes the label independence problem and takes the correlation among labels into account. However, LP suffers two fatal weaknesses. One is the overwhelming time complexity with the increase of the size of label set. The other is the imbalance problem caused by new label sets produced in problem transformation. Pruned datasets and joint decomposition methods are proposed to reduce complexity of the target label set and to deal with the imbalance learning problem.

### 4.1. Multiclass Problem Transformation

**Figure 1:**
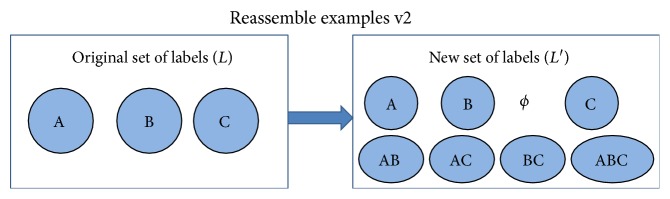
Enumeration for reassembling labels.

There are two steps for transforming the original multilabel classification problem into a multiclass classification problem. First, we get the combination of all the single labels to form a label set *L*, *L* = {*l*_1_, *l*_2_,…, *l*_*n*_}. We use the full enumeration method to reassemble *L* into *L*′, where *L*′ includes all subsets of *L*. Here, each subset denotes a multilabel class label. As an example, the full enumeration method is illustrated in Figure 1. Second, we map each original record by associating it with a new label in *L*′. Algorithm 1 gives the combination label transformation. The notations are described in Table 2. Here, |*L*′| is min(|*D*|, 2^|*L*|^), where 2^|*L*|^ can be calculated by *C*_*n*_^0^ + *C*_*n*_^1^ + ⋯+*C*_*n*_^*n*^, *D* is the training dataset, and *n* denotes the number of the single class labels. It should be noted that we focus on the normal chronic diseases, and the number of single diseases is less than 10.  Table 3 describes the combination of labels from Table 1. After the above two steps, the original multilabel classification problem is transformed into a multiclass classification problem.

### 4.2. Pruned Datasets and Joint Decomposition

We design a pruned dataset method to balance the infrequent label sets. First, we present a threshold to distinguish infrequent label sets from frequent ones for class label records. The records associated with one class label whose size is less than the threshold are decomposed.  Table 4 gives an example of decomposition. Whenever a decomposition operation is executed, the length of the associated combination class label is decreased by one. Meanwhile, the size of all novel class labels would be added to the size of old combination class label. The decomposition is repeated iteratively until all records are frequent, which is described in Algorithm 2, where variable notations are listed in Table 2. Because the threshold would impact the prediction result, it should be traded off between the correctness and accuracy.

To solve the imbalance learning problem, a subset partition method is proposed based on new pruned training datasets. In this work, we present two different strategies. The first one emphasizes the size balance of different class. According to the size of each class label set, the whole training dataset is divided into two or more subsets which are disjoint. The subset partition is described as following. Given a training dataset *T*, $T={\left\{\left(X_{m},{\hat{Y}}_{m}\right)\right\}}_{m=1}^{M}$, where *X*_*m*_ ∈ *χ* ⊂ *R*^*n*^ is the input vector, *χ* is the set of training inputs, ${\hat{Y}}_{m}\in Z\subset R^{K}$ is the desired output, Z is the set of desired outputs, and *M* is the total number of training datasets. Here, *χ*_*j*_∩*χ*_*k*_ = *ϕ*, ⋃_*j*=1_^*N*_*i*_^*χ*_*j*_ = *χ*, with *j*, *k* = 1, *N_i_*, and *j* ≠ *k*. χ*_i_* are the new partition subsets, and *N_i_* is constant, which is determined manually according to all the size of class sets.

**Figure 2:**
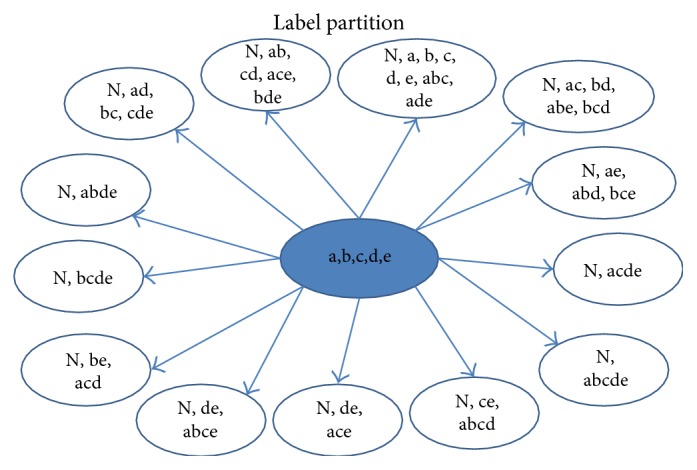
Example of label sets partition.

The second strategy for subset partition focuses on the disjoint of multiclass labels. The idea aims to divide similar labels into different classifiers. First, we calculate the similarity between any two labels. Given a similarity threshold *T_s_*, labels would be divided into different clusters, where labels satisfy the criterion that the similarity is less than the threshold in the same label cluster. An example is given in Figure 2. There are five single class labels to produce 32 multiclass labels. Based on the similarity to perform the subset partition, which is presented in Algorithm 3, 13 labels are formed at last. Here, the similarity threshold is 1/2, which denotes the ratio of the same label number and the maximal label number between two labels.

At last, based on each partitioned training sub dataset, a classifier is constructed separately. Then, it forms several classifiers whose number equals the number of sub datasets. In the prediction, the voting mechanism is adopted to determine the prediction result. The class label which gains the highest prediction probability is the final prediction class label.

### 4.3. Evaluation Metrics

The evaluation of models in multilabel learning needs a special approach because the performance over all labels should be taken into account. In this work, Avg accuracy, precision, recall, and F1 metrics given in (1) are used to evaluate performance of different classification models. Here TP_*i*_, TN_*i*_, FP_*i*_, and FN_*i*_ are true postive, true negative, false positive, and false negative, respectively. The Avg accuracy gives the average per-class effectiveness for one classifier. Precision_micro_ denotes the agreement of the data class labels predicted with those of a classifier calculated from sums of prediction. Recall_micro_ is the effectiveness of a classifier to identify class labels calculated from sums of actual records. F1_micro_ denotes relationships between the positive labels and those given by a classifier based on sums of actual records. Precision_macro_ means an average per-class agreement of the data class labels with those of a classifier given. Recall_macro_ is an average per-class effectiveness of a classifier to identify class labels. F1_macro_ gives the relationships between the positive labels and those given by a classifier based on a per-class average. Given a confusion matrix, these metrics can be computed using the following:

(1) $$\begin{array}{c} Avg\;accuracy=\frac{\sum_{i=1}^{l}\left({TP}_{i}+{TN}_{i}\right)/\left({TP}_{i}+{FP}_{i}+{TN}_{i}+{FN}_{i}\right)}{l}\\ {Precision}_{micro}=\frac{\sum_{i=1}^{l}{TP}_{i}}{\sum_{i=1}^{l}\left({TP}_{i}+{FP}_{i}\right)}\\ {Recall}_{micro}=\frac{\sum_{i=1}^{l}{TP}_{i}}{\sum_{i=1}^{l}\left({TP}_{i}+{FN}_{i}\right)}\\ F1_{micro}=\frac{2{Precision}_{micro}{Recall}_{micro}}{{Precision}_{micro}+{Recall}_{micro}}\\ {Precision}_{macro}=\frac{\sum_{i=1}^{l}{TP}_{i}/\left({TP}_{i}+{FP}_{i}\right)}{l}\\ {Recall}_{macro}=\frac{\sum_{i=1}^{l}{TP}_{i}/\left({TP}_{i}+{FN}_{i}\right)}{l}\\ F1_{macro}=\frac{2{Precision}_{macro}{Recall}_{macro}}{{Precision}_{macro}{\;+\;Recall}_{macro}}. \end{array}$$

## 5. Experiments

### 5.1. Datasets

The real datasets were provided by a medical center. There are 110,300 records of anonymous examination records which include 62 examination items consisting of the basic physical examination items, blood routine examination, liver function test, as well as the diagnosis results marked by the physicians. In this experiment, we focus on 6 normal chronic diseases. They are hypertension, diabetes, fatty liver, cholecystitis, heart disease, and obesity.

We adopt label cardinality (LC) and label density (LD) to describe the datasets. Label cardinality of dataset *D*, denoted by LC(*D*), is the average number of labels of the records in *D*, which is used to quantify the number of the alternative labels. The label density of dataset *D*, denoted by LD (*D*), takes into consideration the number of labels in the classification problem [9]. They are defined in (2), where *D* denotes the dataset of the physical examination records, |*D*| is the size of the dataset *D*, and |*Y*_*i*_| is the number of labels for the *i*th physical record. |*L*| denotes the size of the label set *L*.  Table 5 summarizes the training dataset. It includes the number of records, the number of attributes, and the number of labels, the label cardinality, and the label density. There are two columns for the number of labels. Here, the number of single labels is 6 and the number of combination labels is 64.

(2) $$\begin{array}{c} LC\left(D\right)=\frac{1}{\left|D\right|}\overset{\left|D\right|}{\underset{i=1}{\sum }}\left|Y_{i}\right|\\ LD\left(D\right)=\frac{1}{\left|D\right|}\overset{\left|D\right|}{\underset{i=1}{\sum }}\frac{\left|Y_{i}\right|}{\left|L\right|}. \end{array}$$

### 5.2. The Basic Classification Algorithms

In the experiments, we use three basic classification algorithms, support vector machines (SVM), random forest (RF), and C4.5, to construct classifiers to evaluate the ELPPJD method and compare it with other multilabel classification methods. These classification algorithms are briefly described in this section.

*Support vector machines* (SVM) construct a hyperplane for linear classification or set of hyperplanes in an infinite-dimensional space for nonlinear classification. Suppose that an input *x* can be mapped into some other space of different high dimensionalities. Then, the maximum margin algorithm can be used to construct a separating hyperplane in the new feature space *k*(*x*^*i*^, *x*^*j*^) = *ϕ*(*x*^*i*^)  ·  *ϕ*(*x*^*j*^). The SVM learning algorithm finds a hyperplane (*w*, *b*) such that the quantity $\gamma \underset{i}{\min}y^{i}\left\{<w,\phi \left(x^{i}\right)>-b\right\}$ is maximized, where *<*,*>* denotes an inner product, the vector *w* has the same dimensionality as ϕ, *b* is a real number, and γ is called the margin.

Random forest (RF) combines multiple individual tree decisions to improve prediction accuracy. It was proposed first by Leo Breiman and Adele Cutler [17]. RF consists of multiple classification and regression trees (CART). For every CART, the training datasets are sampled randomly from the total training datasets with replacement, and features are sampled randomly from the total features without replacement. Assuming the total number of features is *M*, then the number of sample features can be close to $\sqrt{M}$, 1/2$\sqrt{M}$, or 2$\sqrt{M}$. RF can be presented as follows: {*h*(*x*, *ϕ*_*k*_), *k* = 1,…}, where ϕ*_k_* are independent, identically distributed random vectors, and each tree casts a unit vote for the most popular class at input *x*.

C4.5 decision tree classification is an algorithm developed by Ross Quinlan [18]. It builds decision trees from a set of training datasets using the concept of information entropy. At each node of the tree, C4.5 chooses the attribute of the data that most effectively splits its set of samples into subsets in one class or the other. The splitting criterion is the normalized information gain. The attribute with the highest normalized information gain is chosen to make the decision. The C4.5 algorithm then recurs on the smaller subsets.

### 5.3. Experiment Setup

The preprocessing of original datasets consists of data cleaning, integration, and transformation, which are conducted by SQL query, MATLAB, and Python, respectively. The experiment platform is based on a CentOS-64 bits Intel (R) Xeon (R) CPU E5-2620 virtual machine with 4 processors and 64GB memory.

**Figure 3:**
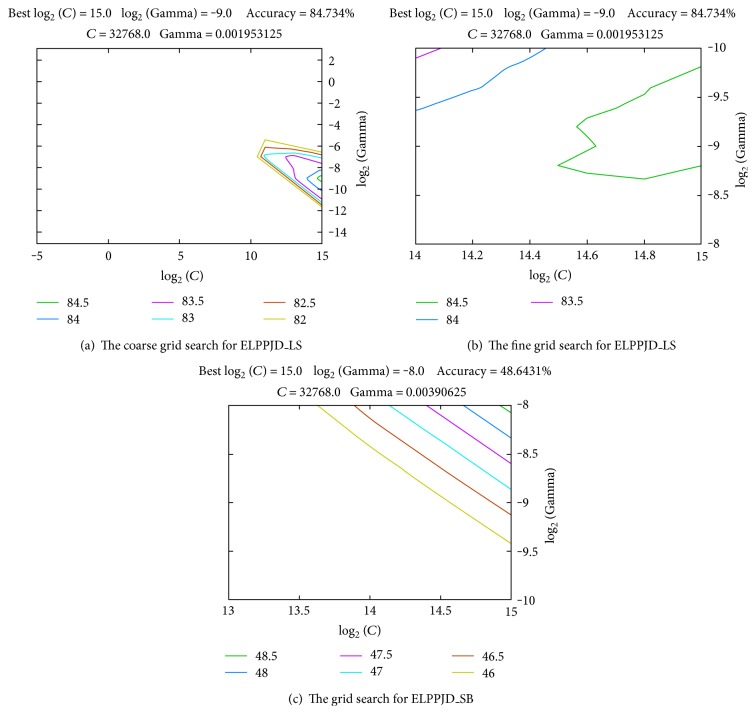
Optimal hyperparameters selected in LIBSVM.

In ELPPJD method, we carry out pruning operation based on the original 64 combination labels dataset and 53 labels are retained. Two subset partition strategies size balanced (SB) and label similarity (LS) are deployed separately. They are denoted by ELPPJD_SB and ELPPJD_LS in the experimental outcome. ELPPJD_SB decomposes the training dataset into 6 subsets, and ELPPJD_LS decomposes it into 8 subsets. We compare ELPPJD with the two outstanding multilabel classification methods named RAkEL and HOMER [15] based on the original training dataset, which includes 110,300 records and 64 combination labels. SVM, RF, and C4.5 are used as the basic multiclass classification algorithms. ELPPJD are carried out based on *LIBSVM* with radial basis function (RFB). RAkEL runs at C4.5 classifier based on *MEKA*. HOMER is executed based on *MULAN* open source algorithms, where RF is the basic classification algorithm. The results of experiments are given in Figure 3, Tables 6–10.

### 5.4. Results and Discussion

Figure 3 shows the selection of the optimal parameters for training models in *LIBSVM.* Two hyperparameters, regularization parameter *c* and a kernel parameter *g*, are tuned by a two-step grid search. First, it is a coarse search. log_2_*c* ∈ {−2,…, 15} and log_2_*g* ∈ {−10,…, 2} are the range of variable parameters with a step of one. There are a total number of 234 combinations of *c* and *g* pairs tuned. An optimal pair (*c*, *g*) is obtained whose value is (15, −9). Second, a fine grid searching is conducted around (*c*, *g*). log_2_*c* ∈ {14,…, 15} and log_2_*g* ∈ {−10,…, −8} with a step of 0.2. Lastly, the final optimal hyperparameter pair (15, −9) is given and the most accuracy rate is 84.734%. Figures 3(a) and 3(c) give the comparison of ELPPJD_SB and ELPPJD_LS. The results show that ELPPJD_LS gives higher accuracy rate.  Table 10 gives further description.

In the experiments, the confusion matrices are used to evaluate the performance of ELPPJD. Based on the result of each prediction, we obtain confusion matrices. When we use ELPPJD_LS, the original physical dataset is decomposed into 8 sub training datasets.  Table 7 shows one ELPPJD_LS outcome, where the sub training dataset consists of 10,828 records and 7 multilabel classes. Label set *L* = {001011, 001100, 010010, 100001, 100110, 111000, 111111}. We evaluate the performance of ELPPJD_LS using the evaluation metrics presented previously in the subsection of evaluation measures, which is given in Table 10.

Random forests as the basic classifier are used to evaluate RAkEL method. We tune the optimal parameters to trade off the performance and resource costs in the RF algorithm. We consider two parameters: max features and n_estimators. Max features denotes the maximum number of features where RF is allowed to try in an individual tree. n_estimators represents the number of trees we want to build. In this experiment, we run random forest on one of the sub training datasets to select the optimal parameters. The averages of accuracy, out of bag errors and time cost are obtained by changing the numbers of selected features and trees.  Table 6 shows that accuracy improves with the increase of the number of trees. However, the time cost grows with the increase of selected features. We consider the accuracy as the primary goal, so the tuple of (15, 80) is chosen as the optimal parameters, which means each RF classifier selects randomly 15 random features and produces 80 trees.

Multilabel classification method RAkEL and HOMER are used to compare with the proposed ELPPJD. In the experiments, RAkEL and HOMER run at the original physical dataset, which includes 110,300 records and 64 multilabel classes. The number of single labels is 6. The basic classifier is C4.5 for RAkEL and RF for HOMER. The parameters are described as following: We tune parameter pair (*k,m*) in RAkEL, where *k* is the number of selected single labels and *m* is the number of combination labels. We select three pairs which consist of (3,15), (4,10), and (5,4). Here, when 3 single labels are selected randomly from the label set, the total number of label combination is 20. We select 15 combination labels to form the training dataset and so on for other two (*k*, *m*) pairs.  Table 8 gives the outcomes with respect to different parameter pairs denoted by RAkEL_k3_m15, RAkEL_k4_m10, and RAkEL_k5_m4. It shows that RAkEL_k3_m15 gives better performance, and it is chosen to compare with ELPPJD in Table 10. In HOMER, parameter *k* denotes the number of child nodes on the first layer. *k* < |*L*|, and here the size of single label set |*L*| = 6. The basic classifier is RF, where max features and n_estimators are set by 15 and 80. The comparison of different *k* is presented in Table 9.

Table 10 shows the performance comparison of ELP-PJD with RAkEL and HOMER based on our training dataset for disease risk prediction. First, the results show that ELPPJD_LS has better performance than ELPPJD_SB. They all adopt pruning and decomposition method to solve the imbalance problem. The difference between ELPPJD_LS and ELPPJD_SB is that the former takes the similarity between labels into account to decompose the training dataset. Second, ELPPJD_LS gives outstanding performance at almost all the metrics than RAkEL and HOMER. The average accuracy reaches 88.59%, which is a good result in multilabel classification.

## 6. Conclusion

We developed an Ensemble Label Power-set Pruned datasets Joint Decomposition (ELPPJD) method to solve the multilabel classification problem for the disease risk prediction. First, we transform the multilabel classification problem into a multiclass classification problem. Then, we propose the pruned datasets and joint decomposition methods to deal with the imbalance learning problem. Two strategies are designed to decompose the training dataset. Experiments are conducted to evaluate the performance of the ELPPJD method. We adopt the 10-fold cross-validation and the metrics consisting of average accuracy, precision, recall, and F-measure. The training dataset includes 62 exam items and 110,300 anonymous patients, 6 types of single diseases, and 64 combination diseases. We contrast two decomposition strategies in ELPPJD. We also compare ELPPJD with two multilabel classification methods RAkEL and HOMER. Results from the experiments show that ELPPJD_LS not only gives better performance than ELPPJDJSB but also outperforms the other two widely used multilabel methods.

## 7. Future Works

For chronic disease prediction, we focus on the following problems in the future work. First, we will develop intelligent mobile applications to provide the service of personalized health risk prediction based on this work. Second, more and more chronic patients use intelligent wearable sensor equipments to monitor the physiological signals; we will collect and analyze the stream data from wearable sensors in real time to make a more accurate health risk assessment. Third, we will apply the result in this work to develop the personalized disease risk prediction models.

## Figures and Tables

**Algorithm 1:**
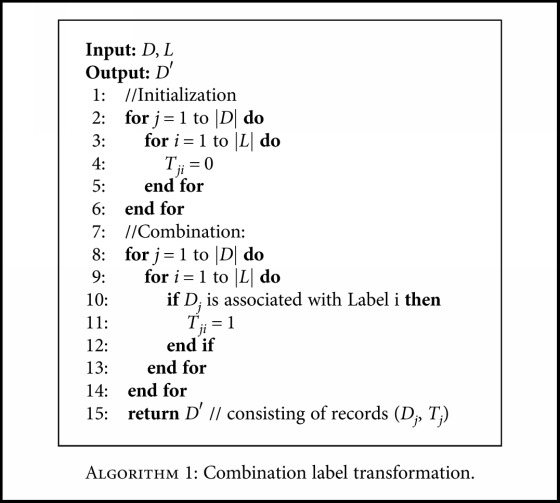
Algorithm 1: Combination label transformation.

**Algorithm 2:**
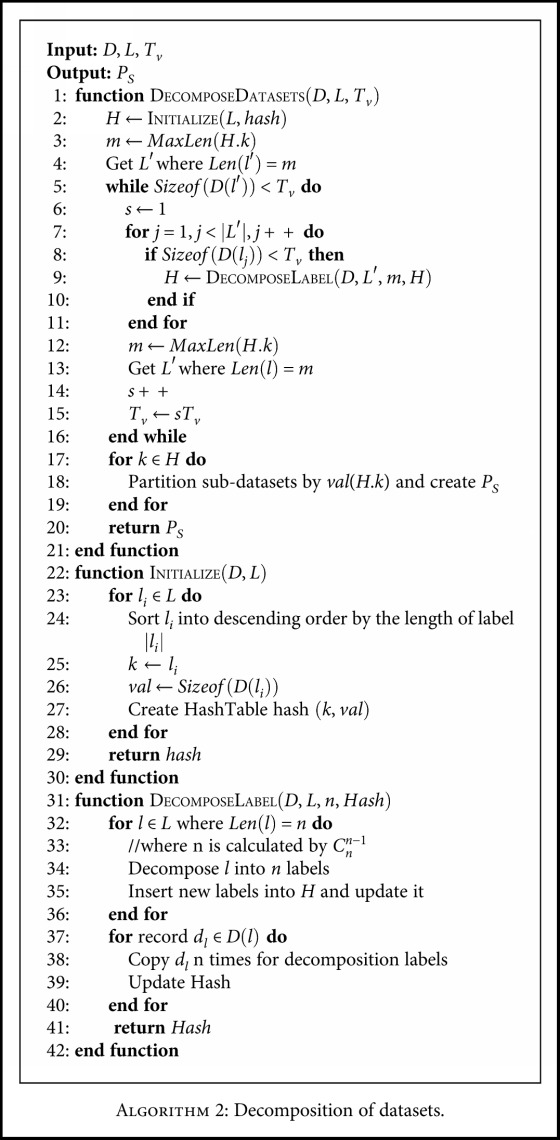
Algorithm 2: Decomposition of datasets.

**Algorithm 3:**
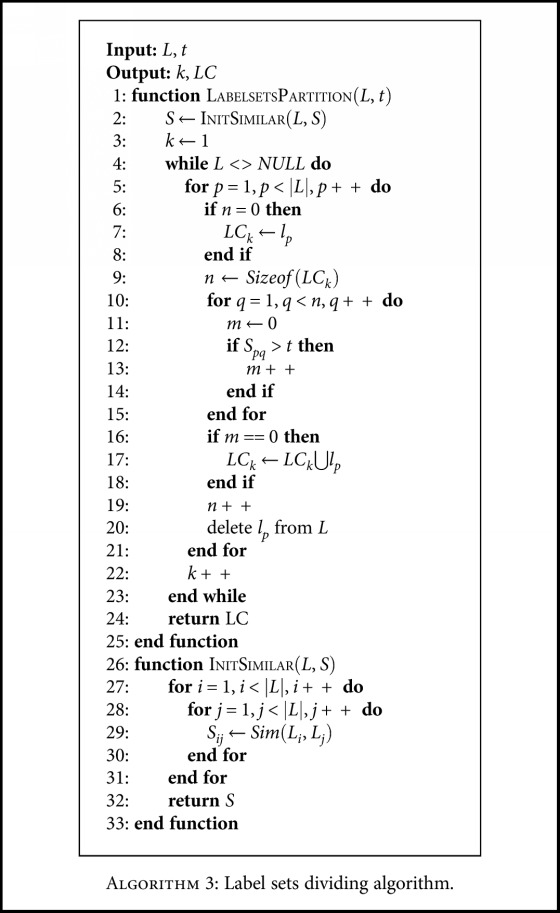
Algorithm 3: Label sets dividing algorithm.

**Table 1:** Multilabel physical examination records description.

Records	Disease a	Disease b	Disease c	Disease d
*r*_1_	^∗^		^∗^	^∗^
*r*_2_	^∗^	^∗^		
*r*_3_		^∗^	^∗^	
…				
*r_m_*		^∗^	^∗^	^∗^

∗ points out the diseases each physical examination record is associated with.

**Table 2:** Variable parameters denotation.

Notation	Denotation
*D*	The training dataset
*L*	Set of class labels
*D′*	The dataset associated with the combination labels
*T_ji_*	The association for the *j*th record to *i*th label, the value is 0 or 1
*l_i_*	The *i*th label item in *L*
*D(l_i_)*	Datasets associated with class label *l_i_*
*T_v_*	Threshold for infrequency records
*P_s_*	Training sub datasets by decomposition
*H*	A Hash function *H*(*k*, val), where *k* is class labels and val is the size of *k* set
*S*	The similarity matrix
*t*	The similarity threshold
*k*	The number of label sets
*LC*	The partition of label sets

**Table 3:** Example of label combination.

Physical records	Combination labels
*r*_1_	acd
*r*_2_	ab
*r*_3_	bc
…	…
*r_m_*	bcd

**Table 4:** Example of label decomposition.

Physical records	Combination labels	Decomposition labels
*r*_1_	acd	ac ad cd

**Table 5:** Description of the multilabel training dataset in the experiments.

Data sets	Records		Attributes	Single labels	Combination labels	Label density	Label cardinality
	Training	Test					
Physical records	99,270	11,030	62	6	64	0.336	2.015

**Table 6:** Random forest classifier parameter tuning on partition training subsets.

NumFeatures	NumTrees	Accuracy	Out of bag error	Time out(s)
10	30	0.9116	0.1483	2.22
10	40	0.9118	0.1473	2.94
10	50	0.9126	0.1466	3.78
10	60	0.9136	0.146	4.51
15	30	0.9175	0.1394	2.88
15	40	0.9167	0.1387	3.88
15	50	0.9185	0.138	4.99
15	60	0.9185	0.1377	5.84
15	70	0.9195	0.1373	6.84
15	80	0.9197	0.1373	7.83
15	100	0.9185	0.1369	9.99
20	30	0.9150	0.137	3.69
20	50	0.9189	0.1354	6.25
20	70	0.9186	0.1346	8.76
30	40	0.9173	0.1347	7.27
30	60	0.9170	0.1342	10.93
40	40	0.9190	0.1343	9.09
40	50	0.9195	0.1338	11.36
40	60	0.9202	0.1334	13.84

**Table 7:** Confusion matrix of ELPPJD_LS based on LIBSVM.

		Prediction						
		001011	001100	010010	100,001	100,110	111,000	111,111
Real class	001011	4048	105	61	127	4	2	89
	001100	24	2622	167	8	79	66	33
	010010	22	97	407	2	5	30	7
	100,001	63	3	7	1038	31	4	36
	100,110	0	30	16	7	707	19	0
	111,000	0	22	26	3	10	374	5
	111,111	15	0	6	19	1	6	405

**Table 8:** RAkEL parameters tuning.

Metrics	RAkEL_k3_m15	RAkEL_k4_m10	RAkEL_k5_m4
Avg accuracy	0.583	0.484	0.547
Precision_micro_	0.543	0.544	0.577
Recall_micro_	NaN	NaN	NaN
F1_micro_	0.744	0.689	0.712
Precision_macro_	NaN	NaN	NaN
Recall_macro_	NaN	NaN	NaN
F1_macro_	0.575	0.578	0.558

**Table 9:** HOMER parameters tuning.

Metrics	HOMER_RF_k2	HOMER_RF_k3	HOMER_RF_k4	HOMER_RF_k5	HOMER_RF_k6
Avg accuracy	0.4755	0.5043	0.5079	0.5152	0.5128
Precision_micro_	0.5483	0.5987	0.6336	0.6639	0.6694
Recall_micro_	0.758	0.7761	0.7030	0.6767	0.6639
F1_micro_	0.6363	0.6759	0.6665	0.6702	0.6666
Precision_macro_	0.5337	0.6042	0.596	0.6045	0.6125
Recall_macro_	0.7442	0.7688	0.6706	0.6409	0.6318
F1_macro_	0.5957	0.6489	0.6070	0.5936	0.5927

**Table 10:** Performance evaluation for different multilabel methods.

Metrics	ELPPJD_SE	ELPPJD_LS	RAkEL_C4.5	HOMER_RF
Avg accuracy	0.516	0.8859	0.583	0.5152
Precision_micro_	0.516	0.8859	0.543	0.6639
Recall_micro_	0.516	0.8859	NaN	0.6767
F1_micro_	0.516	0.8859	0.744	0.6702
Precision_macro_	0.52	0.8082	NaN	0.6045
Recall_macro_	0.5046	0.8603	NaN	0.6409
F1_macro_	0.5122	0.8334	0.575	0.5936
